# Supplementary material for: Predation and spatial connectivity interact to shape ecosystem resilience to an ongoing regime shift
Source: Nat Commun. 2024 Feb 12;15:1304. doi: 10.1038/s41467-024-45713-1 (PMC10861472; doi:10.1038/s41467-024-45713-1)
Supplement: Supplementary file 3 — Reporting Summary [file 41467_2024_45713_MOESM3_ESM.pdf]

nature portfolio

Corresponding author(s): Agnes B Olin
Last updated by author(s): Jan 14, 2024

Reporting Summary

Nature Portfolio wishes to improve the reproducibility of the work that we publish. This form provides structure for consistency and transparency in reporting. For further information on Nature Portfolio policies, see our Editorial Policies and the Editorial Policy Checklist.

Statistics

For all statistical analyses, confirm that the following items are present in the figure legend, table legend, main text, or Methods section.

n/a	Confirmed	
☐	☒	The exact sample size (*n*) for each experimental group/condition, given as a discrete number and unit of measurement
☒	☐	A statement on whether measurements were taken from distinct samples or whether the same sample was measured repeatedly
☐	☒	The statistical test(s) used AND whether they are one- or two-sided<br>*Only common tests should be described solely by name; describe more complex techniques in the Methods section.*
☐	☒	A description of all covariates tested
☐	☒	A description of any assumptions or corrections, such as tests of normality and adjustment for multiple comparisons
☐	☒	A full description of the statistical parameters including central tendency (e.g. means) or other basic estimates (e.g. regression coefficient) AND variation (e.g. standard deviation) or associated estimates of uncertainty (e.g. confidence intervals)
☐	☒	For null hypothesis testing, the test statistic (e.g. *F*, *t*, *r*) with confidence intervals, effect sizes, degrees of freedom and *P* value noted<br>*Give P values as exact values whenever suitable.*
☒	☐	For Bayesian analysis, information on the choice of priors and Markov chain Monte Carlo settings
☐	☒	For hierarchical and complex designs, identification of the appropriate level for tests and full reporting of outcomes
☐	☒	Estimates of effect sizes (e.g. Cohen's *d*, Pearson's *r*), indicating how they were calculated

Our web collection on statistics for biologists contains articles on many of the points above.

Software and code

Policy information about availability of computer code

Data collection	No software was used to collect the data.
Data analysis	R version version 4.2.1 was used for data analysis, ArcGIS Pro 2.4 and QGIS 3.18 were used for some of the spatial calculations. All code is available at https://github.com/agnesolin/stickleback-wave and at doi.org/10.5281/zenodo.10473335. Key R-packages are referenced in the text with version numbers (sdmTMB 0.3.0, glmmTMB 1.1.7, DHARMa 0.4.6, mctest 1.3.1). Additional R-packages that were used for smaller subtasks can be found in the referenced code.

For manuscripts utilizing custom algorithms or software that are central to the research but not yet described in published literature, software must be made available to editors and reviewers. We strongly encourage code deposition in a community repository (e.g. GitHub). See the Nature Portfolio guidelines for submitting code & software for further information.

Data

Policy information about availability of data

All manuscripts must include a data availability statement. This statement should provide the following information, where applicable:

- Accession codes, unique identifiers, or web links for publicly available datasets
- A description of any restrictions on data availability
- For clinical datasets or third party data, please ensure that the statement adheres to our policy

The underlying environmental data are not shared publicly due to restrictions on data sharing for some of the datasets, but we are happy to discuss requests

addressed to the corresponding author. The processed data (juvenile fish density data and associated data on drivers for each data point) are available at github.com/agnesolin/stickleback-wave and at doi.org/10.5281/zenodo.1047333586. The majority of the juvenile fish density data are available in the Swedish national database for coastal fish monitoring (slu.se/kul), sea surface temperature data were sourced from the Copernicus Baltic Sea L4 dataset (https://doi.org/10.48670/moi-00156) and seal count data were downloaded from the Swedish database for environmental monitoring data (www.sharkweb.smhi.se). Source data for Figs. 1a, 2 and 3 are provided with this paper.

Research involving human participants, their data, or biological material

Policy information about studies with human participants or human data. See also policy information about sex, gender (identity/presentation), and sexual orientation and race, ethnicity and racism.

Reporting on sex and gender	The study did not involve human participants
Reporting on race, ethnicity, or other socially relevant groupings	The study did not involve human participants
Population characteristics	The study did not involve human participants
Recruitment	The study did not involve human participants
Ethics oversight	The study did not involve human participants

Note that full information on the approval of the study protocol must also be provided in the manuscript.

Field-specific reporting

Please select the one below that is the best fit for your research. If you are not sure, read the appropriate sections before making your selection.

☐ Life sciences ☐ Behavioural & social sciences ☒ Ecological, evolutionary & environmental sciences

For a reference copy of the document with all sections, see nature.com/documents/nr-reporting-summary-flat.pdf

Ecological, evolutionary & environmental sciences study design

All studies must disclose on these points even when the disclosure is negative.

Study description	The study seeks to characterise an ongoing shift in dominance from large predatory fish (northern pike, Esox lucius; Eurasian perch; Perca fluviatilis) to three-spined stickleback (Gasterosteus aculeatus) along the Swedish Baltic Sea coastline, and why some areas are more resistant to the shift. To characterise how absolute and relative densities have changed over time we made use of a collation of juvenile surveys carried out at the end of summer. The surveys were carried out as part of a range of monitoring and research programmes, and do not as such comprise a coordinated effort. No data were collected specifically for the present study. In total, we made use of >7000 samplings collected between 2001 and 2020, between 55 and 60.5 degrees north.<br><br>To capture drivers of the observed patterns we made use of a range of environmental variables, often after processing the data to suit our hypotheses (e.g. calculating degree days from the temperature data). These datasets are described in detail in the manuscript. The focus below is mainly on the juvenile fish surveys.<br><br>The data were analysed using generalised linear mixed models. The first set of models included main effects of offshore stickleback densities, wave exposure, distance from the open sea, as well as an interaction between offshore stickleback densities and distance from the open sea. Year-specific random spatial effects were also included. The second set of models included main effects of offshore stickleback densities, wave exposure, distance from the open sea, connectivity, predation from seals and cormorants, fishing pressure and temperature, as well as interactions between offshore stickleback densities and distance from the open sea, between connectivity and predation, between connectivity and fishing and between temperature and distance from the open sea. Year was included as a random effect. Three different response variables were used: relative predator dominance (N = 3491), absolute predator densities (N = 7415) and absolute stickleback densities (N = 7167).
Research sample	Each sample consisted of estimated density (number of individuals per sample, which represents roughly 80 m2) of young-of-the-year (age 0+) Eurasian perch (Perca fluviatilis), northern pike (Esox lucius) and three-spined stickleback (Gasterosteus aculeatus). As juvenile and adult sticklebacks can be difficult to tell apart in the field, these were added together (absolute majority will be juveniles at this time of the year). The fish were not sexed but the sample will contain both sexes.<br><br>As the study was based completely on previously collected data, we could not steer the sampling choice. We included all available data collected 55-60.5 degrees north (to correspond to the Baltic Proper, an area coherent in terms of management and ecology) after 2000, when the stickleback increase started. The sample is meant to represent the fish community along the coastline of the Baltic Proper after 2000. Most of the data, which were collected within a range of monitoring and research programmes, are available in the Swedish national database for coastal fish monitoring (slu.se/kul).
Sampling strategy	No statistical method was used to determine sample size; instead we included as many of the existing datasets on juvenile fish densities as possible. Even with the data exclusions as described below, this resulted in a sample size of >7000 with good spatio-

	temporal spread. The different monitoring programmes and research studies that contributed to the dataset will have had different purposes and sampling strategies. All included data were collected towards the end of summer, and would have been designed to estimate juvenile density in a given area. The choice of area, the number of samples taken, and the number of years of sampling will have differed between studies and monitoring programmes depending on the purpose of the work.
Data collection	Young-of-the-year juvenile coastal fish were sampled using low-impact pressure waves; a standard method in Baltic Sea juvenile fish monitoring (see /doi.org/10.1016/j.jembe.2006.12.008 for a detailed description). In short, an underwater detonation stunned or killed all small fish (2-20 cm) with a swim bladder within the blast radius (ca. 5m using the current detonation standard). The fish were then collected, identified and counted. The data were generally recorded using pen and paper and eventually transferred to the Swedish national database for coastal fish monitoring (slu.se/kul). The data were collected by multiple fish monitoring programs and research projects conducted during the past 20 years. In most cases, there was no information available on who (individuals) performed the sampling. The work was carried out mainly by the Swedish University of Agricultural Sciences, the Swedish Board of Fisheries, Stockholm University, the county boards along the Swedish Baltic Sea coast and the consultancies/organisations Naturvatten i Roslagen AB, JP Aquakonsult, Upplandsstiftelsen, Sveriges Vattenekologer AB, Hushållningssällskapet Rådgivning Nord AB and Hydrophyta.
Timing and spatial scale	The juvenile surveys were carried out during summer (in most cases, July-September) between 2001 and 2020. Spatially, the sampling covered ca 680 km stretch of the western Baltic Sea coastline (55-60.5 degrees north). Some locations were sampled in multiple years, and some only for one year.
Data exclusions	Some data were excluded in order to achieve a more coherent and robust dataset. We excluded data prior to 2000 (as samples were very scarce) and data above 60.5 degrees north (to focus in on an area which is more coherent in terms of both ecology and management). Further, we excluded data collected outside July-September, as they may not well represent juvenile fish composition in summer (e.g. the stickleback already migrating offshore). Finally, we excluded data from furthest into the archipelago as there were so few data datapoints here, thus having an undue influence on the effect of some of the identified relationships. However, these deep-archipelago samples followed our identified patterns well (>95 % predator-dominated).
Reproducibility	There were no experimentation involved. We have made all code and the final data frame used for the statistical analysis available so that the analysis can be reproduced.
Randomization	There was no allocation into experimental groups.
Blinding	Since all data was collected using field surveys, no blinding was possible.

Did the study involve field work? ☒ Yes ☐ No

Field work, collection and transport

Field conditions	The study is based on already collected data over a long time period and a large area, involving many projects and programmes. The field conditions will thus have varied a lot, but any possible effects are likely to be largely averaged out as a result of the large sample size.
Location	The data used in the large-scale analyses were sampled in shallow coastal bays situated along the Swedish Baltic Sea coastline (55-60.5 degrees north). The coastline runs in a N-S direction. Average water depth was just under 2 m.
Access & import/export	All sampling was carried out by certified personnel with necessary permits, in compliance with the EU Directive 2010/63/EU and national legislation. The main scientific surveys included in the dataset were covered by Permit 2007-0883 issued to the Swedish Board of Fisheries by the Swedish Animal Welfare Agency and Permit C 139/13 issued to the Swedish University of Agricultural Sciences by the Ethical Committee on Animal Experiments.
Disturbance	The method used to sample juvenile fish (underwater detonations) stuns and/or kills all small fish (2-20cm) with a swim bladder, but minimises disturbance caused to benthic habitats (see /doi.org/10.1016/j.jembe.2006.12.008).

Reporting for specific materials, systems and methods

We require information from authors about some types of materials, experimental systems and methods used in many studies. Here, indicate whether each material, system or method listed is relevant to your study. If you are not sure if a list item applies to your research, read the appropriate section before selecting a response.

Materials & experimental systems

n/a	Involved in the study	
☒	☐	Antibodies
☒	☐	Eukaryotic cell lines
☒	☐	Palaeontology and archaeology
☐	☒	Animals and other organisms
☒	☐	Clinical data
☒	☐	Dual use research of concern
☒	☐	Plants

Methods

n/a	Involved in the study	
☒	☐	ChIP-seq
☒	☐	Flow cytometry
☒	☐	MRI-based neuroimaging

Animals and other research organisms

Policy information about studies involving animals; ARRIVE guidelines recommended for reporting animal research, and Sex and Gender in Research

Laboratory animals	The study did not involve laboratory animals.
Wild animals	The study focused on young-of-the-year (age 0+) fish from three species: Eurasian perch (Perca fluviatilis), northern pike (Esox lucius) and three-spined stickleback (Gasterosteus aculeatus). The fish were sampled using low-impact pressure waves induced by underwater detonations; a standard method in Baltic Sea monitoring of coastal fish recruitment (see /doi.org/10.1016/j.jembe.2006.12.008 for a detailed description). In short, an underwater detonation generates a pressure wave which stuns or kills all small fish with a swim bladder (2-20cm length). The fish are collected (by swing nets from boat and/or by snorkelers) and then identified and counted on the boat. The individual fish were not sexed. Injured individuals were directly euthanized using the (at the time) recommended and approved methods (MS-222, destroying the brain, breaking the neck). Since we did not conduct most of the historical sampling included in the data extracted from the national database, we cannot describe exactly how the fish were euthanized in each respective sampling.<br><br>For our covariates, we also used previously collected count data on grey seals and great cormorants
Reporting on sex	The fish were not sexed.
Field-collected samples	The sampled fish were released live or (if injured) euthanized in the field, using approved methods (see above).
Ethics oversight	All sampling was carried out by certified personnel with necessary permits, in compliance with the EU Directive 2010/63/EU and national legislation. The main scientific surveys included in the dataset were covered by Permit 2007-0883 issued to the Swedish Board of Fisheries by the Swedish Animal Welfare Agency and Permit C 139/13 issued to the Swedish University of Agricultural Sciences by the Ethical Committee on Animal Experiments.

Note that full information on the approval of the study protocol must also be provided in the manuscript.
